# Dietary bile acid supplementation influences protein digestibility and alters plasma amino acid profiles in broiler chickens fed conventional and reduced-protein diets

**DOI:** 10.1016/j.psj.2025.105107

**Published:** 2025-03-29

**Authors:** Yumin Bao, Shemil P. Macelline, Peter H. Selle, Peter V. Chrystal, Mengzhu Wang, Sonia Y. Liu, AiZhi Cao, Mehdi Toghyani

**Affiliations:** a Redox Pty Ltd, Minto, NSW 2566, Australia; b School of Life and Environmental Sciences, Faculty of Science, The University of Sydney, Sydney NSW 2006, Australia; c Poultry Research Foundation, The University of Sydney, Camden NSW 2570, Australia; d Aviagen, Auckland, New Zealand; e Shandong Lachance, China

**Keywords:** Bile acids, Plasma amino acid, Reduced protein diets, Protein digestibility

## Abstract

Reduced crude protein (CP) diets based on wheat have been shown to lower body weight (BW), increase feed conversion ratio (FCR), and elevate fat-pad deposition in broiler chickens. Bile acids facilitate lipid digestion and nutrient absorption by emulsifying fats and promoting micelle formation in the intestine. Therefore, it was hypnotized that supplementing reduced CP diets with bile acids may enhance fat utilization, improve energy efficiency, and mitigate the negative effects of low-CP diets on broiler performance and nutrient digestibility. Four dietary treatments were arranged in a 2 × 2 factorial layout, with two CP levels (standard and a 30 g/kg CP reduction), with or without 0.2 g/kg bile acids. A total of 840 off-sex male Ross 308 chicks were randomly assigned into 24 floor pens, with six replicates of 35 birds per treatment. From 0 to 42 days post-hatch, there was no interaction between dietary CP and bile acids supplementation for BW, feed intake (FI), FCR, abdominal fat pad, and nutrient digestibility (*P* > 0.05). As a main effect, reducing dietary CP decreased BW, FI, fat digestibility, and increased FCR and abdominal fat pad (*P* < 0.01). The reduced CP diet resulted in lower plasma concentrations of valine, isoleucine, arginine, histidine, phenylalanine, leucine, and tryptophan, while increasing lysine, methionine, and threonine concentrations (*P* < 0.01). Bile acids supplementation enhanced dry matter and protein digestibility (*P* < 0.05). Bile acids also showed a tendency to increase plasma methionine concentrations (*P* = 0.055). A significant interaction effect was observed for overall mortality, with bile acids supplementation reducing mortality in birds fed reduced CP diets (*P* < 0.01). These findings suggest that in reduced CP diets, the current ideal AA profile may be deficient in arginine and histidine, potentially contributing to increased fat pad weight. Elevated wheat-derived non-starch polysaccharide concentrations might lower fat digestibility in reduced CP diets. Supplementation of bile acids in these diets could mitigate endogenous taurine losses and improve overall health in broiler chickens.

## Introduction

When diets contain the optimal balance of amino acids, a modest decrease of 20 to 30 g/kg in dietary crude protein (CP) has been found to sustain the performance and carcass yields of broiler chickens. However, reducing CP by more than 30 g/kg has been reported to negatively affect bird performance and lead to higher accumulation of adipose fat (Kidd et al., 2021). The reduced CP levels are usually achieved by increasing non-bound amino acids and feed grains such as wheat inclusions at the expense of soybean meal. Consequently, this increases the starch and non-starch polysaccharides (NSP) levels and concurrently reduces added fat as the dietary energy source. Such changes in the dietary structure of reduced CP diets have been reported to increase relative fat-pad weight and FCR, although ileal starch and essential amino acids (AA) digestibility may improve (Crystal et al., 2020). In contrast, Moss et al. (2018) reported that the relatively high starch and NSP concentration in reduced CP diets improved starch digestibility but significantly reduced AA digestibility. Broiler chickens are highly sensitive to water-soluble NSP, with lipid digestibility being most adversely affected by its concentration (Maisonnier et al., 2003).

Bile acids are synthesized in the liver from cholesterol through a complex series of enzymatic reactions, resulting primarily in the formation of cholic acid and chenodeoxycholic acid (Russell, 2003). These primary bile acids are then conjugated with the amino acids glycine or taurine to form bile salts, which are secreted into the bile, stored in the gallbladder, and subsequently released into the duodenum upon feeding (Chiang, 2009). In the small intestine, bile salts play an essential role in emulsifying dietary lipids, thereby facilitating the digestion and absorption of fats and fat-soluble vitamins. Certain intestinal bacteria express bile salt hydrolase enzymes that deconjugate the bile salts, liberating free primary bile acids (Joyce and Gahan, 2016). Subsequently, specific bacterial species, such as *Clostridium scindens*, can further metabolize these primary bile acids into secondary bile acids like deoxycholic acid and lithocholic acid through processes including dehydroxylation. These microbial transformations not only modify the physicochemical properties of bile acids but also influence host physiology, including lipid metabolism and gut microbiome composition (Ridlon et al., 2014). Furthermore, bile acids exert direct antimicrobial effects by disrupting bacterial cell membranes, thereby suppressing the growth of potentially pathogenic bacteria such as *Clostridium perfringens* and *Escherichia coli* (Hofmann and Eckmann, 2006; Wahlström et al., 2016). These antimicrobial properties apply selective pressure on the gut microbial community, often leading to an enrichment of bile-tolerant taxa such as *Lactobacillus, Bacteroides*, and *Bilophila* (Ridlon et al., 2014).

A majority of bile salts are reabsorbed in the ileum and returned to the liver via enterohepatic circulation, but a portion escapes reabsorption and enters the large intestine (Ridlon et al., 2006). Young birds are limited in their capacity to recycle bile acids efficiently, resulting in decrease in the pool of bile salts (Zaefarian et al., 2019). Furthermore, elevated dietary concentration of NSPs particularly in wheat, has been reported to enhance faecal bile acid loss in human (Walters et al., 1975), rats (Overton et al., 1994) and poultry (Choct, 1999; Matin et al., 2016). Traditionally, bile acids have been primarily recognised for their role in promoting lipid and fat-soluble vitamin absorption. However, recent studies have shown that bile acids are pleiotropic, hormone-like signalling molecules that also regulate mucosal homeostasis and inflammation (Chen et al., 2019). In addition, an *in vitro* study indicates that bile acids influence the digestion and assimilation of dietary protein by enhancing the hydrolytic activity of pancreatic proteases (Gass et al., 2007).

Therefore, given the potential of bile acids to enhance fat and protein digestibility as well as exert antimicrobial effects, this study aimed to investigate whether supplementing exogenous bile acids in reduced crude protein diets based on wheat, characterised by elevated NSP contents, could mitigate the performance losses previously observed.

## Materials and methods

### Animal ethic statement

The feeding study was performed according to the specific guideline approved by the Animal Ethics Committee of the University of Sydney (2020/1764).

### Experimental design and diet preparation

A total of 840 off-sex male Ross 308 chicks were reared from 0 to 42 days post-hatch. Starter, grower and finisher diets were offered from 0 to14, 15 to 28, 29 to 42 days post-hatch, respectively. Using a completely randomized experimental design, four experimental diets based on wheat, soybean meal and canola meal were arranged in a 2 × 2 factorial layout to evaluate the effect of two dietary levels of crude protein (standard and reduced by 30 g/kg) and two levels of bile acids supplementation (0 and 0.2 g/kg feed). Each of the four treatment was replicated 6 times in floor pens with 35 birds per replicate. The standard positive control diets were formulated to meet or exceed the 2019 Aviagen Ross 308 nutrition specifications and reduced CP diets were formulated with around 30 g/kg CP reduction as shown in Table 1. Nitrogen corrected apparent metabolizable energy (AMEn) of the diets was calculated from the AMEn of the dietary ingredients and each ingredients contribution to AMEn pool, and the standardized ileal digestible (SID) amino acids of the diets was calculated from the SID of different amino acids of the dietary ingredients and each ingredients contribution to SID pool of relevant amino acids based on the principles of least-cost linear programming (Pesti and Miller, 1993). The bile acids were obtained from Shandong Lachance Animal Health Care Co. Ltd, China, which contained 30 % active total bile acids. Celite (Celite World Minerals,2500 San Miguelito Rd Lompoc, CA), a source of acid-insoluble ash (AIA) was added to the finisher diets as an inert dietary marker for measuring nutrients digestibility. All diets contained commercially relevant amounts of phytase (1000 FTU/kg) and xylanase (100 g/tonne) and were steam-pelleted at 80°C. Birds were initially fed diets in crumble form from 0 to 14 days post-hatch, and thereafter in pellet form from 15 to 42 days post-hatch.

**Table 1:** Composition and calculated nutrient specifications of positive control diets (PC) and reduced protein diets (RC). Values in parentheses are analyzed values.

Ingredients (g/kg)	Starter		Grower		Finisher	
	PC	RC^1^	PC	RC	PC	RC
Wheat	585	660	632	764	611	754
Dextrose	-	15.2	-	-	-	-
Soybean meal	278	194	282	133	252	115
Canola meal	75	30	30	30	40	30
Soybean oil	19	7.0	22	4.0	50	28
L-Lysine HCl	4.3	7.7	3.2	7.5	2.5	6.6
L-Methionine	3.4	4.6	2.9	3.9	2.4	3.4
L-Threonine	2.0	3.6	1.3	3.2	0.9	2.7
L-Valine 98 %	0.4	2.4	-	2.1	-	1.5
L-Arginine	0.8	4.1	-	3.9	-	3.2
L-Tyrosine	-	2.0	-	1.8	-	1.1
L-Iso-leucine 98 %	0.3	2.2	-	2.0	-	1.6
L-Leucine 98.5 %	-	2.7	-	2.3	-	1.5
L-Histidine	-	0.5	-	0.4	-	0.2
Glycine	-	2.1	-	3.1	-	2.9
Salt	1.1	-	1.5	-	1.8	-
Sodium bicarbonate	2.5	4.3	2.1	4.2	1.7	4.2
Potassium carbonate	0.0	3.9	-	3.5	-	3.9
Limestone flour	14.3	16.3	10.8	11.4	8.6	9.2
MDCP┼	10.9	14.9	8.0	9.0	6.2	7.3
Xylanase	0.1	0.1	0.1	0.1	0.1	0.1
Phytase	0.1	0.1	0.1	0.1	0.1	0.1
Choline chloride 60 %	0.8	0.8	0.7	0.7	0.6	0.6
Celite^TM^	0.2	20.0	0.2	7.6	20.2	20.2
Premix╪	2.0	2.0	2.0	2.0	2.0	2.0
*Nutrients*						
AMEn (MJ/kg)	12.0	12.0	12.4	12.4	12.9	12.9
Protein (g/kg)	253 (258)	225 (227)	240 (246)	210 (213)	225 (221)	195 (196)
Fat (g/kg)	38.3 (34.7)	25.4 (22.6)	41.6 (38.4)	23.1 (22.3)	68.4 (62.9)	46.7 (42.3)
SID⸹ Lys (g/kg)	13.4	13.4	12.1	12.1	10.8	10.8
SID Met + Cys (g/kg)	10.0	10.0	9.1	9.1	8.4	8.4
SID Thr (g/kg)	9.0	9.0	8.1	8.1	7.2	7.2
SID Trp (g/kg)	2.7	2.1	2.6	1.9	2.4	1.8
SID Ile (g/kg)	9.0	9.0	8.5	8.2	7.9	7.5
SID Leu (g/kg)	15.2	14.8	14.7	13.3	13.8	11.9
SID Arg (g/kg)	14.4	14.4	13.0	12.9	12.2	11.6
SID Val (g/kg)	10.1	10.1	9.3	9.1	8.8	8.2
SID His (g/kg)	5.3	4.7	5.1	4.2	4.8	3.8
SID Glu (g/kg)	45.0	41.8	47.3	41.5	44.1	39.7
SID ASP (g/kg)	16.0	12.6	16.5	10.4	15.0	9.5
SID Gly equivalent (g/kg)	15.5	14.5	15.0	14.6	14.1	13.7
Total Ca (g/kg)	10.9 (8.8)	10.8 (9.2)	8.7 (7.4)	8.7 (6.9)	7.5 (6.2)	7.5 (6.5)
Total P (g/kg)	6.5 (6.8)	6.4 (7.1)	5.4 (6.1)	5.1 (5.8)	4.9 (5.6)	4.6 (5.5)
Available P (g/kg)	5.2	5.6	4.4	4.4	4.0	4.0
Na (g/kg)	1.8 (1.5)	1.8 (1.4)	1.8 (1.4)	1.8 (1.6)	1.8 (1.6)	1.8 (1.5)
K (g/kg)	10.1 (11.4)	10.1 (10.6)	9.8 (10.7)	8.9 (10.4)	9.1 (9.7)	8.7 (9.6)
DEB mEq/kg	280	280	273	250	256	250

┼ MDCP: Monodicalcium phosphate.

╪ The premix contained (per kg of diet): [IU] retinol, 12,000; cholecalciferol, 5,000; [mg] tocopherol, 50; menadione, 3; thiamine, 3; riboflavin, 9; pyridoxine, 5; cobalamin, 0.025; niacin, 50; pantothenate, 10; folate, 2; biotin, 0.2; copper, 20; iron, 40; manganese, 110; cobalt, 0.25; iodine, 1; molybdenum, 2; zinc, 90; selenium, 0.3.

⸹ SID**:** standardized ileal digestible amino acids concentration**s**.

### Bird management

Birds had unlimited access to feed and water in an environmentally controlled deep litter facility on the Camden Campus of the University of Sydney (NSW 2570). The temperature was maintained at 32°C for the first 3 days and then gradually reduced to 21°C by the third week. The lighting program followed breeder recommendations (Aviagen, 2022), starting with 23 h of light for the first three days, gradually reduced to 18 h by the end of the grower phase and further to 16 h by the end of the finisher phase. Body weights were measured at day 0, 14, 28, and 42, and feed intake was recorded at similar intervals to calculate feed conversion ratios (FCR). The occurrence of mortality or culling was documented daily, and the body weights of deceased birds were used to correct the FCR for mortality.

### Sample collection and chemical analysis

On day 42, 6 birds selected at random from each pen were euthanized to collect distal ileal digesta and carcass yield data, including *pectoralis* major and minor muscles (breast fillet and tenderloin, respectively), leg quarter (skin off and bone in) and fat pad. Distal ileal samples were taken from below the mid-point between Meckel's diverticulum and the ileo-caecal junction. Digesta were pooled per pen and freeze-dried. The nitrogen content of both diets and digesta samples was analyzed using a 0.25-gram sample in a combustion analyzer (Leco Corporation, St. Joseph, MI), with ethylenediaminetetraacetic acid (EDTA) used as a calibration standard.

Crude protein content was calculated by multiplying the percentage of nitrogen by a correction factor (6.25), following the method described by Siriwan et al. (1993). The dry matter and fat content of diets and digesta were determined using methods specified by AOAC, 2006. Crude protein, fat, and dry matter digestibility were calculated using the following equation:

$$\text{Nutrients}\;\text{digestibility}\;\text{coefficient}\;\left(\%\right)=\;\frac{\left(\text{Nutrien}t_{\text{diet}}/\text{AI}A_{\text{diet}}\right)-\left(\text{Nutrien}t_{\text{digesta}}/\text{AIA}{\;}_{\text{digesta}}\right)}{\left(\text{Nutrien}t_{\text{diet}}/\text{AI}A_{\text{diet}}\right)}\times \;100$$

Duplicate samples from each diet (on an as-fed basis) were collected and then analyzed for mineral concentration using an inductively coupled plasma-optical emission spectrometer (ICP-OES) (Agilent, Mulgrave, Victoria, Australia), following the methodology outlined by Maxfield and Mindak (1985).

On day 34, blood samples were collected from the wing vein of three randomly selected birds per pen (18 birds per treatment). Blood samples were then centrifuged, and the resulting plasma samples were stored at −80°C prior to analysis. The concentrations of twenty proteinogenic amino acids in the plasma samples were determined using precolumn derivatization amino acid analysis with 6-aminoquinolyl-N-hydroxysuccinimidyl carbamate (AQC; Waters™ AccQTag Ultra; Waters Corporation Milford, MA). The derivatives were separated and quantified using reversed-phase ultra-performance liquid chromatography (UPLC), with all amino acids detected based on UV absorbance.

### Statistical analyses

The dataset was assessed for normality and then analyzed using a 2-way ANOVA within the GLM procedure in JMP®13 (SAS Institute Inc., JMP Software, Cary, NC), to determine the main effects of crude protein (CP) level, bile acid supplementation, and their 2-way interaction, based on the following statistical model:

$$Y_{ijk}=\mu +\alpha_{i}+\beta_{j}+{\left(\alpha \beta \right)}_{ij}+\varepsilon_{ijk}$$

Where Y_ijk_ is the k^th^ observation at the i^th^ CP level and j^th^ bile acid supplementation

μ is the overall mean response

α_i_​ is the effect of the i^th^ level of CP

β_j_​ is the effect of the j^th^ level of bile acid supplementation

(αβ)_ij_​ is the interaction effect between CP level and bile acid supplementation

ε_ijk_​ is the residual error term

Each pen served as an experimental unit, and the values reported in the tables represent means with pooled standard error of the mean (SEM). If a significant treatment effect was identified, differences between treatments or main effects were determined using least square differences tests. Statistical significance was defined at *P* < 0.05, and trends were discussed for *P* < 0.1.

## Results

The effects of dietary treatments on growth performance are shown in Table 2. In the starter period, birds receiving the RC diets recorded a lower body weight (*P* = 0.003). Bile acid supplementation reduced weight gain during the starter phase (390 versus 373 g/bird, *P* = 0.032). In addition, a significant interaction between dietary CP and bile acids supplementation resulted in increased FCR in birds receiving the reduced CP diets only when diets were supplemented with bile acids (*P* = 0.037). However, over the grower period, this interaction resulted in an improved FCR of the birds fed the reduced CP diet supplemented with bile acids compared to non-supplemented diets (*P* = 0.038). As a main effect, CP reduction decreased both BWG (*P* < 0.001) and FI (*P* = 0.003) over the grower and finisher phases. There was no interaction between dietary CP and bile acids supplementation on growth performance in finisher phase. Over the entire 42-day production period, CP reduction resulted in decreased BWG (*P* = 0.001) and FI (*P* = 0.003) and increased FCR (*P* = 0.001), while bile acid supplementation improved the FCR (*P* = 0.027). There was a significant interaction between dietary CP levels and bile acids supplementation on the overall mortality rate where adding bile acids to reduced CP diets significantly reduced the mortality rate from 4.3 % to 0.95 % (*P* = 0.003).

**Table 2:** Effects of dietary treatments on growth performance from 0 to 14, 15-28, 29-42, and 0- 42 days post-hatch.

Treatments		Starter d 0-14			Grower d 15-28			Finisher d 29-42			Overall d 0-42			Mortality %
CP	Bile acids	BWG	FI	FCR	BWG	FI	FCR	BWG	FI	FCR	BWG	FI	FCR	
		g/bird	g/bird	g/g	g/bird	g/bird	g/g	g/bird	g/bird	g/g	g/bird	g/bird	g/g	
PC	Nil	399	471	1.180^b^	1274	1734	1.369^b^	1847	2900	1.571	3520	5109	1.451	1.429^bc^
RC	Nil	388	460	1.197^b^	1203	1715	1.430^a^	1778	2788	1.568	3361	4957	1.475	4.286^a^
PC	Plus	380	458	1.174^b^	1291	1739	1.367^b^	1831	2832	1.547	3510	5026	1.439	3.333^ab^
RC	Plus	357	443	1.243^a^	1221	1694	1.387^b^	1770	2783	1.573	3348	4920	1.469	0.952^c^
SEM		7.4	8.8	0.012	13.8	15.9	0.009	17.4	23.6	0.008	28.4	37.7	0.004	0.790
*Main effect*														
CP levels														
	PC	394^a^	465	1.177	1283^a^	1741^a^	1.368	1819^a^	2866^a^	1.559	3515^a^	5067^a^	1.445^b^	2.231
	RC	368^b^	452	1.222	1212^b^	1724^b^	1.408	1774^b^	2785^b^	1.570	3355^b^	4938^b^	1.472^a^	2.619
Bile acids														
	Nil	390^a^	465	1.189	1239	1729	1.399	1812	2844	1.569	3441	5033	1.463^a^	2.875
	Plus	373^b^	451	1.208	1256	1716	1.377	1800	2808	1.560	3429	4973	1.454^b^	2.143
Source of variation (*P*-values)														
CP levels		0.003	0.148	0.001	<0.001	0.032	<.001	0.002	0.003	0.158	<0.001	0.003	<0.001	0.766
Bile acids		0.032	0.117	0.098	0.210	0.450	0.029	0.522	0.141	0.146	0.700	0.127	0.027	0.377
Bile × CP		0.422	0.849	0.037	0.994	0.582	0.038	0.814	0.192	0.092	0.957	0.548	0.345	0.003

Each value for each treatment represents the mean of 6 replicates of 35 birds each.

a-g Means within a column not sharing a superscript differ significantly at the *P* < 0.05 level for the treatment effects and at the P level shown for the main effects. PC: positive control; RC: reduced crude protein; BWG: body weight gain; FI: feed intake.

Table 3 showed the effects of dietary treatments on carcass yield, and ileal digestibility of dry matter (DM), CP and crude fat. Dietary CP reduction significantly reduced yields of breast meat and increased relative fat pad weight (*P* = 0.001). Bile acids supplementation did not influence carcass yield parameters. Reducing dietary CP significantly reduced ileal crude fat digestibility (*P* = 0.001) but increased DM digestibility (*P* = 0.013). However, there was no significant effect of bile acids or its interaction with CP levels for fat digestibility although bile acids supplementation numerically increased fat digestibility in the conventional diets (*P* > 0.05). Regardless of dietary CP levels, bile acids supplementation significantly increased CP (*P* = 0.012) and DM (*P* = 0.001) digestibility by an average of 6.26 and 11.3 %, respectively.

**Table 3:** Effects of dietary treatments on carcass yield and ileal digestibility of dry matter (DM), CP and fat.

Treatments			Yield (g/100 g)			Ileal digestibility coefficient		
CP	Bile acids	*p. major*	*p. minor*	Leg quarter	Fat pad	DM	CP	Fat
PC	Nil	20.1	3.65	21.3	0.91	0.656	0.781	0.969
RC	Nil	19.1	3.40	21.4	1.22	0.666	0.783	0.955
PC	Plus	20.3	3.64	21.2	0.93	0.727	0.820	0.980
RC	Plus	19.3	3.37	21.3	1.15	0.745	0.843	0.948
SEM		0.250	0.044	0.130	0.045	0.005	0.007	0.006
*Mail effect*								
CP levels								
	PC	20.2^a^	3.65^a^	21.3	0.92^b^	0.690^b^	0.800	0.969^a^
	RC	19.2^b^	3.38^b^	21.3	1.18^a^	0.705^a^	0.813	0.955^b^
Bile acids								
	Nil	19.6	3.65	21.3	1.06	0.661^b^	0.782 b	0.962
	Plus	19.8	3.38	21.3	1.04	0.736 a	0.831^a^	0.964
Source of variation (*P*-values)								
CP levels		< 0.001	<0.001	0.649	< 0.001	0.013	0.089	0.001
Bile acids		0.503	0.703	0.451	0.606	<0.001	<0.012	0.775
Bile × CP		0.974	0.877	0.877	0.275	0.376	0.141	0.158

Each value for each treatment represents the mean of 6 birds per replicate, and 6 replicates per treatment.

a-b Means within a column not sharing a superscript differ significantly at the *P* < 0.05 level for the treatment effects and at the P level shown for the main effects. PC: positive control; RC: reduced crude protein.

Table 4, Table 5 summarize the effect of dietary treatments on plasma amino acids profile. Reducing dietary CP significantly decreased plasma concentrations of Arginine (Arg), Histidine (His), Isoleucine (Ile), Cysteine (Cys), Phenylalanine (Phe), Tryptophan (Trp), Valine (Val), Asparagine (Asn), Aspartate (ASP), Glutamine (Glu), and Serine (Ser), but elevated plasma concentration of Methionine (Met), Threonine (Thr), Glutamine (Glu) and Glycine (Gly) (*P* < 0.05). Bile acids supplementation tended (*P* = 0.055) to increase plasma concentration of Met, irrespective of dietary CP levels.

**Table 4:** Effects of dietary treatments on plasma non-essential and total amino acids concentration (µg/ml).

Treatments		Amino acids										
CP	Bile acids	Ala	Asn	Asp	Cys	Gln	Glu	Gly	Pro	Ser	Tyr	TAA
PC	Nil	54.4	22.3	8.49	15.4	173.6	20.5	54.7	65.2	59.7	27.8	805
RC	Nil	55.4	17.9	6.51	15.4	229.3	18.8	65.1	64.5	54.6	26.8	853
PC	Plus	56.1	26.2	7.54	15.1	186.7	22.9	54.8	69.3	59.9	30.1	845
RC	Plus	57.1	18.8	6.93	14.2	241.1	18.8	65.9	64.3	56.8	27.7	873
SEM		3.331	1.303	1.009	0.457	8.477	1.214	1.738	2.478	1.412	1.816	18.18
*Main effect*												
CP levels												
	PC	55.3	24.2^a^	8.02	15.3^a^	180.2^b^	21.7^a^	54.7^b^	67.3	59.8^a^	28.9	825^b^
	RC	56.3	18.3^b^	6.72	14.3^b^	235.2^a^	18.8^b^	65.5^a^	64.4	55.7^b^	27.3	863^a^
Bile acids												
	Nil	54.9	20.1	7.5	14.9	201.5	19.6	59.9	64.8	57.1	27.3	829
	Plus	56.6	22.4	7.2	14.6	213.9	20.9	60.3	66.8	58.3	28.9	859
Source of variation (*P*-values)												
CP levels		0.763	0.002	0.250	0.042	<0.001	0.027	<0.001	0.263	0.009	0.375	0.049
Bile acids		0.622	0.08	0.813	0.562	0.157	0.309	0.798	0.433	0.411	0.383	0.117
Bile × CP		0.998	0.260	0.539	0.917	0.944	0.330	0.845	0.382	0.491	0.696	0.585

Each value for each treatment represents the mean of 3 birds per replicate, and 6 replicates per treatment.

a-b Means within a column not sharing a superscript differ significantly at the *P* < 0.05 level for the treatment effects and at the P level shown for the main effects. PC: positive control; RC: reduced crude protein.

**Table 5:** Effects of dietary treatments on plasma essential and total amino acids concentration (µg/ml).

Treatments		Amino acids									
CP	Bile acids	Arg	His	Ile	Leu	Lys	Met	Phe	Thr	Trp	Val
PC	Nil	80.7	13.7	14.9	24.3	31.2	15.8	19.2	70.7	4.32	28.7
RC	Nil	61.8	6.43	12.6	19.2	38.2	20.5	15.9	96.3	3.31	25.9
PC	Plus	83.1	13.8	15.6	25.5	34.6	17.1	19.7	73.3	4.44	29.4
RC	Plus	68.8	6.11	12.6	18.9	36.9	21.4	15.7	92.5	3.25	25.6
SEM		6.617	0.778	0.629	2.041	2.252	0.536	0.378	3.081	0.136	0.936
*Main effect*											
CP levels											
	PC	81.8^a^	13.8^a^	15.3^a^	24.9^a^	32.9^b^	16.4^b^	19.5^a^	72.0^b^	4.38^a^	29.1^a^
	RC	65.3^b^	6.28^b^	12.6^b^	19.0^b^	37.5^a^	20.9^a^	15.8^b^	94.4^a^	3.28^b^	25.8^b^
Bile acids											
	Nil	71.3	10.1	13.8	21.7	34.7	18.1	17.6	83.5	3.81	27.3
	Plus	75.9	10.0	14.1	22.2	35.7	19.2	17.7	82.9	3.84	27.5
Source of variation (*P*-values)											
CP levels		0.021	<0.001	<0.001	<0.001	<0.001	<0.001	<0.001	<0.001	<0.001	0.002
Bile acids		0.489	0.916	0.634	0.586	0.646	0.055	0.735	0.847	0.825	0.851
Bile × CP		0.731	0.740	0.601	0.381	0.308	0.647	0.360	0.302	0.509	0.595

Each value for each treatment represents the mean of 3 birds per replicate, and 6 replicates per treatment.

a-b Means within a column not sharing a superscript differ significantly at the *P* < 0.05 level for the treatment effects and at the P level shown for the main effects. PC: positive control; RC: reduced crude protein.

## Discussion

Overall, broiler performance surpassed the targets for male Ross 308 broilers, and birds in this study gained higher weight and exhibited lower FCR than the breeder's standard (Aviagen, 2022). However, reducing 30 g/kg CP significantly compromised body weight gain, feed intake and FCR. These results contradict previous findings by van Harn et al. (2019), where reducing CP by 30 g/kg in wheat-maize-soy diets supplemented with Lys, Met, Thr, Val, Ile, Arg, Trp, and Gly improved BWG and FCR from 11 to 35 days post-hatch. This study utilized wheat as the grain source. Chrystal et al., 2020 reported that maize-based diets perform better than wheat-based diets under reduced CP conditions. Thus, the lower BWG and higher FCR observed in this study may be partially attributed to the use of wheat as the primary grain. In van Harn et al.'s study (2019), the apparent faecal digestibility of amino acids (AFD AA) was used to balance the AA profile, with AFD ratios of Val, Ile, Arg, Trp, and Gly+Ser to Lys being 80 %, 71 %, 114 %, 22 %, and 150 %, respectively. In contrast, the present study employed the standardised ileal digestibility of AA (SID AA) to balance the profile. In reduced CP diets, the SID ratios of Val, Ile, Arg, Trp, and Gly to Lys were 76 %, 69 %, 107 %, 16 %, and 127 %, respectively. This resulted in higher plasma concentrations of Lys, Met + Cys, and Thr, and lower concentrations of Val, Ile, Arg, His, Trp, and Gly + Ser compared to the normal CP diet. The higher Lys, Met and Thr concentration in plasma may indicate that in the reduced CP diets, Lys, Met, and Thr are not utilized efficiently, likely due to deficiencies of other amino acids, leading to their elevated plasma levels. This imbalance in the AA profile at the site of protein synthesis likely contributed to the birds' compromised performance (Waldroup et al., 1976). Despite formulating the reduced CP diets to achieve a similar ideal AA ratio as the PC diets, it appears that either the ideal AA ratio or the rates of digestion and absorption of AA in the reduced CP diets differ from those in normal CP diets. Beyond Lys, Met, and Thr, Val is the fourth limiting amino acid in reduced CP diets. Its deficiency strongly depresses feed intake, even when other amino acid requirements are met (Maynard et al., 2022). Based on plasma Val concentration in PC and reduced CP diets, the ideal SID Val to Lys ratio could be maintained at 80 %. Recently Maynard et al. (2022) demonstrated that in reduced CP diets, dietary Arg and His deficiency significantly increased fat-pad weight. Arginine is crucial for lipid metabolism, with its deficiency leading to increased fat deposition. Studies show that broilers on Arg-deficient diets have higher abdominal fat, while Arg supplementation reduces fat by modulating lipid metabolism (Zampiga et al., 2018). Similarly, His is essential for protein synthesis and muscle growth. Though its effect on fat pad weight is less clear, His deficiency can impair growth and carcass traits, potentially altering body composition (Moro et al., 2020). The plasma Arg and His levels in current PC diets suggest that the optimal SID Arg:Lys and His:Lys ratios should exceed 110 % and 40 %, respectively. Increasing the ideal SID Trp to Lys ratio from 17 % to 22 % has been shown to significantly improve FCR (Maynard et al., 2022). Similarly, based on plasma Trp levels in PC diets, the optimal ratio in this study is 22 %.

For non-essential amino acids, glutamate (Glu) plus glutamine (Gln) and aspartate (Asp) plus asparagine (Asn) are often reported as glutamic acid (GLU) and aspartic acid (ASP) in the literature (Li et al., 2011). Dietary CP reduction significantly lowered plasma Asn concentration, suggesting that the SID ASP to Lys ratio of 139 % in the conventional diet may be sufficient, while CP reduction could result in ASP deficiency. In reduced CP diets, the SID GLU, Ala, and Pro to Lys ratios decreased to 368 %, 44 %, and 124 %, respectively, but this did not affect their plasma concentrations. This indicates that the depressed performance in reduced CP diets is unlikely related to dietary GLU, Ala, or Pro levels. Reduced crude protein (CP) diets based on maize showed better growth performance compared to wheat-based diets. Reducing CP by 57 g/kg in maize-soy diets increased BWG by 7 % from 7 to 35 days post-hatch, whereas the same CP reduction in wheat-soy diets impaired growth performance (Chrystal et al., 2020). The ASP to Lys ratio in maize-soy and wheat-soy-based diets was 86 % and 60 %, respectively. While ASP may be deficient in reduced CP wheat-based diets, it is unlikely to have contributed to the compromised growth performance observed in this study. Limited research exists on ASP requirements in broilers. Corzo et al., 2005 demonstrated that in reduced CP (40 g/kg) maize-based diets supplemented with all essential amino acids, adding ASP restored BWG from 5 to 21 days post-hatch, with an ASP to Lys ratio of approximately 165 %. This is significantly higher than the 122 % ratio recommended by Texas A&M University (Wu, 2014). In general, maintaining an ASP to SID Lys ratio above 100 % appears optimal for reduced CP diets, regardless of whether they are based on wheat or maize.

In this study, reduced CP diets significantly decreased ileal crude fat digestibility, likely due to increased dietary NSP content, as expected. However, bile acid supplementation did not improve fat digestibility in reduced CP diets, suggesting that insufficient bile acids may not be the cause. The higher soluble viscous arabinoxylan content in wheat-based reduced CP diets could reduce gut motility, potentially decreasing the diffusion and transportation of emulsions, mixed micelles, fatty acids, and bile acids in the small intestine (Meng et al., 2004). In contrast, the higher fat content in conventional diets may enhance fat digestibility, possibly by increasing gut motility (Golian and Maurice, 1992). It remains unclear whether reduced fat digestibility in this study resulted from increased NSPs or lower dietary fat content. Additionally, lower dietary fat concentrations may have influenced the efficacy of bile acid supplementation. Interestingly, bile acid supplementation significantly improved protein digestibility, irrespective of dietary CP levels. Bile acid supplementation tended to increase plasma Met concentration in both conventional and reduced CP diets, despite reduced CP diets already elevating plasma Met levels (*P* = 0.055). Since taurine is synthesized from Met and Cys metabolism (Han et al., 2020) and constitutes a major component of endogenous AA loss through bile in broiler chickens (Ravindran, 2021), the improved CP digestibility observed may be attributed to a reduction in endogenous bile secretion or taurine utilization. These findings align with previous *in vitro* research showing that bile acid supplementation enhances protein digestibility by accelerating hydrolysis during digestion by pancreatic proteases (Gass et al., 2007). While increased lipase activity has been reported in response to bile acid supplementation in broiler chickens (Lai et al., 2018), direct upregulation of protease activity by bile acids hasn't been studied in poultry. In reduced CP diets, improved DM digestibility is likely driven by increased starch digestibility and lower feed intake (Chrystal et al., 2022; Moss et al., 2018), while reduced fat digestibility may contribute to poorer performance, as observed in the present study. Although bile acid supplementation enhanced DM digestibility, it did not affect fat digestibility, suggesting the improvement in DM digestibility may be linked to better CP digestibility.

Notably, adding bile acids to reduced CP diets significantly decreased the mortality rate. In low CP diets, the liver experiences heightened metabolic demands for protein synthesis and deamination. Additionally, the higher NSP content in such diets can lead to increased faecal loss of bile acids, placing additional strain on the liver to produce more bile acids. Therefore, supplementing exogenous bile acids may have relieved metabolic stress on the liver and enhanced the survivability of the birds. In young broiler chickens, liver is responsible for 90 to 95 % de novo fatty acid synthesis (Griffin et al., 1992). Excessive lipid accumulation in the liver (hepatic steatosis) can cause severe biological damage, impairing fat processing, protein production, and hormone and toxin regulation, potentially leading to fatal outcomes. If untreated, hepatic steatosis may progress to fibrosis, cirrhosis, or liver failure (Fellenberg and Speisky, 2006). Additionally, taurine has been shown to relieve oxidative stress and lipid-related metabolic disorders (Han et al., 2020). Thus, exogenous bile acid supplementation may have reduced metabolic pressure on the liver, alleviated oxidative stress, and improved overall bird health.

## Conclusions

These findings indicate that the ideal AA profile and the rates of AA digestion and absorption in reduced CP diets may differ from those in standard CP diets, particularly in wheat-based formulations. Optimizing both AA composition and utilization efficiency is essential to support bird performance under reduced CP conditions. Based on plasma amino acid concentrations, the following ideal SID AA-to-Lys ratios may be appropriate for reduced crude protein diets to support improved performance: Met+Cys 76 %, Thr 68 %, Val 80 %, Ile 72 %, Arg 110 %, Trp 22 %, His 44 %, and Gly-equivalent 120 %. Ensuring adequate dietary Arg and His may help reduce abdominal fat deposition under these conditions.

The higher NSP content in wheat-based, low-fat reduced CP diets may impair fat digestibility and contribute to reduced performance. Although bile acid supplementation at 0.2 kg/ton did not improve fat digestibility in the present study, it may help mitigate endogenous taurine loss, reduce metabolic load on the liver, and consequently enhance broiler chicken health. Further research is warranted to evaluate the effects of bile acid supplementation in maize-based versus wheat-based reduced CP diets. A limitation of this study was the lack of direct assessment of bile acid supplementation and dietary crude protein effects on biomarkers of gut health, which could provide deeper insights into the mechanisms underlying the observed performance outcomes.

## CRediT authorship contribution statement

**Yumin Bao:** Investigation, Methodology, Data curation, Formal analysis, Writing – original draft. **Shemil P. Macelline:** Data curation, Formal analysis, Writing – review & editing. **Peter H. Selle:** Methodology, Writing – review & editing. **Peter V. Chrystal:** Methodology, Writing – review & editing. **Mengzhu Wang:** Data curation, Formal analysis, Writing – review & editing. **Sonia Y. Liu:** Conceptualization, Methodology, Funding acquisition, Writing – review & editing. **AiZhi Cao:** Conceptualization, Methodology, Funding acquisition, Writing – review & editing. **Mehdi Toghyani:** Formal analysis, Project administration, Supervision, Writing – review & editing.

## Disclosures

We declare that we have no financial and personal relationships with other people or organizations that can inappropriately influence our work, and there is no professional or other personal interest of any nature or kind in any product, service and/or company that could be construed as influencing the content of this paper.
